# Quantitative Microplate Assay for Real-Time Nuclease Kinetics

**DOI:** 10.1371/journal.pone.0154099

**Published:** 2016-04-21

**Authors:** Jonas Eriksson, Ülo Langel

**Affiliations:** Department of Neurochemistry, Stockholm University, Stockholm, Sweden; Tulane University, UNITED STATES

## Abstract

Utilizing the phenomenon of nucleases exposing oligonucleotide phosphate backbones to phosphatases we present a novel quantitative method for kinetics of nuclease catalysis. Inorganic phosphate released from nuclease products by phosphatases could be quantified in real-time by a fluorescent sensor of inorganic phosphate. Two different nucleases were employed, showing the versatility of this assay for multiple turnover label-free nuclease studies.

## Introduction

Nucleases are indispensable to all organisms. They are essential for the DNA replication machinery, removing primers, proofreading etc. Nucleases play integral roles in recombination and repair of the genomic DNA. Many cell defense mechanisms include nucleases; the RISC complex degrading specific RNAs, restriction endonucleases cleaving foreign double-stranded DNA (dsDNA) and the CRISPR-Cas systems storing information about invading viruses in the genome of many bacteria and archaea [1–3].

Nucleases have been an important addition to the molecular biology toolbox for decades. Especially important has been the applicability of type II restriction endonucleases on recombinant techniques, allowing scientists to ‘cut-and-paste’ different dsDNA substrates together [4]. Other examples of common uses of nucleases are biosensors [5], removal of specific nucleic acid species [6] and also genomic editing (ex. CRISPR-Cas) [7].

There is a plethora of different nucleases acting on different substrates with different modes of action. A multitude of assays for determining enzymatic activities of nucleases have been developed; mostly by incorporating fluorescent- or radioactive labels in the substrate oligonucleotides [8,9]. Major drawbacks can be seen in these assays such as: the inability to perform the nuclease reaction under multiple turnover conditions [10], requiring labeling of substrate and/or enzyme, low sensitivity and discontinuity. Assays without modifications generally require high concentrations of substrate and enzyme as the read-outs have low sensitivity, such as running samples in gels or measuring absorbance [11]. Many assays are discontinuous which can cause faulty assumptions as a lot of the reaction data is missed between time-points and there is increased labor compared to continuous assays [12,13]. Nuclease assays often measure either the amount of substrate left or they measure the cleavage of substrate as a yes/no parameter even though many nucleases cleave each substrate molecule several times, causing significant information loss [14].

Nucleosides are chained together by phosphodiesters as a backbone to form oligonucleotides. When a nuclease cleaves an oligonucleotide the phosphodiester backbone connecting two nucleosides is transformed to either a 5’ or 3’ phosphomonoester or 2’, 3’-cyclic phosphodiester bound to one of the products. Nuclease cleavage of the phosphodiester backbone exposes the phosphate for phosphatases to act upon, hydrolyzing the phosphate into inorganic phosphate (P_i_). Using this knowledge an assay is presented here where various nucleases can be used together with natural, label-free substrates. Briefly, the assay principle is as follows: Nuclease cleaves oligonucleotide substrate, producing products with exposed phosphates, these are in turn released by a phosphatase as P_i_ which thereafter is quantified in real-time by a fluorescent phosphate sensor. This reaction can be measured continuously using nanomolar concentrations of substrate.

Products formed by nucleases are mainly oligonucleotides or nucleosides with 5’ or 3’ monophosphates. Alkaline phosphatases have long been known to efficiently cleave as 5’ and 3’ phosphomonoester substrates [15]. FastAP is an improved alkaline phosphatase capable of quickly cleaving phosphates from DNA, RNA and nucleotides and it is active in a wide variety of buffers. However, alkaline phosphatases are incapable of cleaving 2’, 3’-cyclic phosphodiesters which are formed by several ribonucleases and also RNA-cleaving ribozymes and deoxyribozymes (DNAzymes) [16]. T4 polynucleotide kinase (T4PNK) has the ability to cleave 2’, 3’-cyclic phosphodiesters, an ability which has been naturally evolved to thwart the innate immune response of the bacterium which the T4 phage infects; thus, T4PNK can replace alkaline phosphatase when the nuclease reaction form 2’,3’-cyclic phosphodiester products [17].

P_i_ concentrations have been measured by discontinuous low sensitivity assays such as reacting P_i_ with molybdate to form colored complexes [18]. A modern assay utilized phosphate binding protein of *E*. *coli* coupled to 7-diethylamino-3-[N-(2-maleimidoethyl)carbamoyl]coumarin (MDCC-PBP) as a biosensor for P_i_ allowing real-time P_i_ concentration determination with high sensitivity as P_i_ binding produces a 6- to 8-fold increase in fluorescence [19].

To show the versatility of this assay two different nucleases are used. Exonucleases are represented by exonuclease III from *E*. *coli* (ExoIII) while endonucleases are represented by a ‘10–23’ DNAzyme [20]. The choice of these two enzymes also show the applicability of this assay for both protein- and nucleic acid-based nucleases.

## Materials and Methods

All oligonucleotides were synthesized and purified by ThermoFisher (Table 1). DNA oligonucleotides were ordered with desalting and RNA oligonucleotides as HPLC purified. Oligonucleotides were diluted in RNase- and DNase-free ultrapure water to 10 μM working stocks. The water used was produced by purifying ddH_2_O using a Milli-Q Advantage A10 Water Purification System equipped with a BioPak Polisher (Merck Millipore). The purified water had undetectable levels of P_i_. Tris amino Ultra-pure (Angus Chemical Company). Sodium chloride (Sigma Aldrich). Magnesium chloride hexahydrate was from Riedel-de Haën. Hydrochloric acid for setting Tris buffer pH (Scharlau). MDCC-PBP (commercial name Phosphate Sensor), T4 polynucleotide kinase, FastAP and Exonuclease III (ThermoFisher). MDCC-PBP was kept as 5 μM stocks at -80°C. Purine nucleoside phosphorylase (PNPase) and 7-methylguanosine (7-MEG) (Sigma) were kept as stocks at -80°C of 500 U/ml and 30 mM respectively. Reagents with high P_i_ background levels were pretreated with 0.001 U/ml PNPase and 200 μM 7-MEG at RT until P_i_ background was less than 100 nM. 96 well half area black plates with clear flat bottom and NBS^™^-coating (Corning) were chosen as assay plates to minimize binding of reagents to the plate. The plates were highly contaminated by P_i_, this was solved by rinsing the plates with P_i_-free water and centrifugation upside down to remove the water. After centrifugation each plate was covered with a plastic adhesive seal (MidSci). Flexstation II multiplate reader with 8-channel microfluidics was from Molecular Devices.

**Table 1:** Sequences of oligonucleotides.

	Sequence
**DNAzymes and substrate**	
DzSJ^[^^20^^]^	5’-GCA CCC AGG CTA GCT ACA ACG ACT CTC TC-3’
I-DzSJ^[^^10^^]^	5’-GCA CCC AGG CTA **C**CT ACA ACG ACT CTC TC-3’
DzSJ RNA substrate^[^^20^^]^	*5’-GGA GAG AGA | UGG GUG CG-3’*
**ExoIII substrate**	
Sense strand	5’-GGT GTT GGA ATT CGC CTT AG-3’
Antisense strand	3’-CCA CAA CCT TAA GCG GAA TC-5’

Ribonucleotides are displayed in italics; Underlined letters mark the 10–23 DNAzyme catalytic loop; Bold letters are inactivating G-C mutations in catalytic loop; | indicate 10–23 DNAzyme cleavage site.

### Assay- and compound plate setups

ExoIII assay plate wells contained 1.5 nM (0.04 U/μl) ExoIII and 1 μM MDCC-PBP in 1X ExoIII buffer (66 mM Tris-HCl pH 7.5, 0.66 mM MgCl_2_). Compound plate contained varying concentrations of substrate together with FastAP (final concentration 0.0004 U/μl) in 1X ExoIII buffer. The plates were incubated inside the Flexstation II for 30 min at 37°C prior to reaction initiation for the plate to reach the desired temperature.

For the DNAzyme reactions the assay plate was set up containing 0.3 U/μl T4PNK, 2 μM RNA substrate and 1 μM MDCC-PBP in 1X DNAzyme buffer (40 mM Tris-HCl pH 7.5, 20 mM MgCl_2_, 50 mM NaCl). The compound plate contained DNAzyme of varying concentrations in 1X DNAzyme buffer. The plates were left to incubate inside the Flexstation for 15 min at RT for equilibration.

The different setups is only due to minimizing the loss of expensive material as the compound plate demands a larger volume, leading to loss of material, for efficient pipetting. To initiate the reactions 10 μl (1/5^th^ of the final reaction volume) from each compound plate well was added to each respective well in the assay plate through the Flexstation fluidics module. Reactions were measured every 5 s over the course of the experiment. ExoIII reactions were measured for 20 minutes after initiation while DNAzyme reactions were measured for 30 minutes as this reaction was slower. Flexstation II settings: λ_Ex_ = 426 nm, λ_Em_ = 466 nm, λ_cutoff_ = 435 nm, reads/well = 30, Photomultiplier tube voltage = High.

### Data analysis

All experiments were repeated three times and each time-point show error bars representing standard error of the mean between the three experiments. Each experiment was normalized to a control reaction containing no substrate and then normalized to the initial fluorescence to remove differences in background fluorescence between each reaction. The fluorescence was then changed to P_i_ concentration through normalization to a standard curve made for the MDCC-PBP in each respective reaction buffer (S5 and S6 Figs). The slope of the linear part of each curve was used as initial velocity (v_0_). The Michaelis constant (K_M_) is defined in eq (1) by the rate constants for enzyme-substrate binding and dissociation (k_on_ and k_off_ respectively) together with the turnover number (k_cat_) or the apparent turnover number (${\text{k}}_{\text{cat}}^{\text{app}}$) which is used for ExoIII. The reverse reaction of ligation is disregarded due to the fact that no evidence has been found for the occurrence of ligation in ExoIII reactions and the reverse reaction rate constant of 10–23 DNAzymes is much smaller than k_cat_. K_M_ and maximal velocity (V_max_) were determined by curve-fitting in GraphPad Prism (GraphPad software) using eq (2). k_cat_ was calculated by dividing V_max_ with initial enzyme concentration [21].

$${\text{K}}_{\text{M }}=\;\frac{{\text{k}}_{\text{off}}\;+\;{\text{k}}_{\text{cat}}}{{\text{k}}_{\text{on}}}$$ (1)

$${\text{v}}_{\text{0}}\;=\;\frac{{\text{V}}_{\text{max}}\;[\text{S}]}{{\text{K}}_{\text{M}}\;+\;[\text{S}]}$$ (2)

## Results and Discussion

General nuclease assays depend on labels and/or separating products by size. The assay presented here uses the phenomenon that when nucleases cleave oligonucleotide backbone phosphodiesters are exposed either in the form of nucleoside monophosphates and/or shorter oligonucleotides with 5’ or 3’ phosphomonoesters or 2’, 3’-cyclic phosphodiesters. The exposed phosphates are in turn released from the product by an alkaline phosphatase or T4PNK, which are present in excess over substrate to prevent the dephosphorylation from being rate-limiting, resulting in the formation of P_i_, which in turn binds to MDCC-PBP thereby inducing a conformational change. This MDCC-PBP conformational change causes an increase in fluorescence of the environment-sensitive fluorophore MDCC. Downstream reactions of coupled enzyme assay can’t be allowed to be rate-limiting. To keep the nuclease reaction as rate-limiting, and thus represent the measured kinetic rates, an excess of phosphatase and MDCC-PBP over substrate was used.

### P_i_ background

Contamination of reagents and water by P_i_ is common and poses a problem for phosphate-sensing assays. To remove any background P_i_ from reagents they were treated with PNPase and 7-MEG [22]. PNPase catalyzes the simultaneous cleavage and phosphorylation of nucleosides (eg. 7-MEG), resulting in a nucleobase and a ribose-1-phosphate as products. However, addition of PNPase could potentially introduce a negative effect on the readout of the nuclease assay results where the perceived increase in P_i_ concentration would be lower than expected. An experiment was set up to determine the maximal allowed concentration of PNPase in the nuclease reactions. The concentration deemed to be high enough to remove background P_i_ from reagents at RT overnight but not high enough to significantly interfere with the assay was 0.0002 U/ml PNPase which has a reaction rate of 0.002 nM s^-1^ at 37°C (S1 Fig).

### ExoIII kinetics

**Fig 1:**
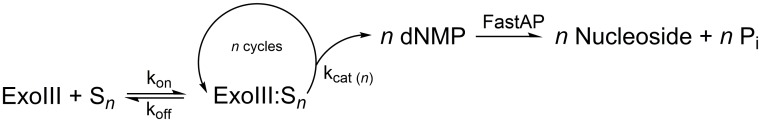
Kinetic model of Exonuclease III hydrolysis of a dsDNA substrate with *n* nucleotides susceptible to hydrolysis.

**Fig 2:**
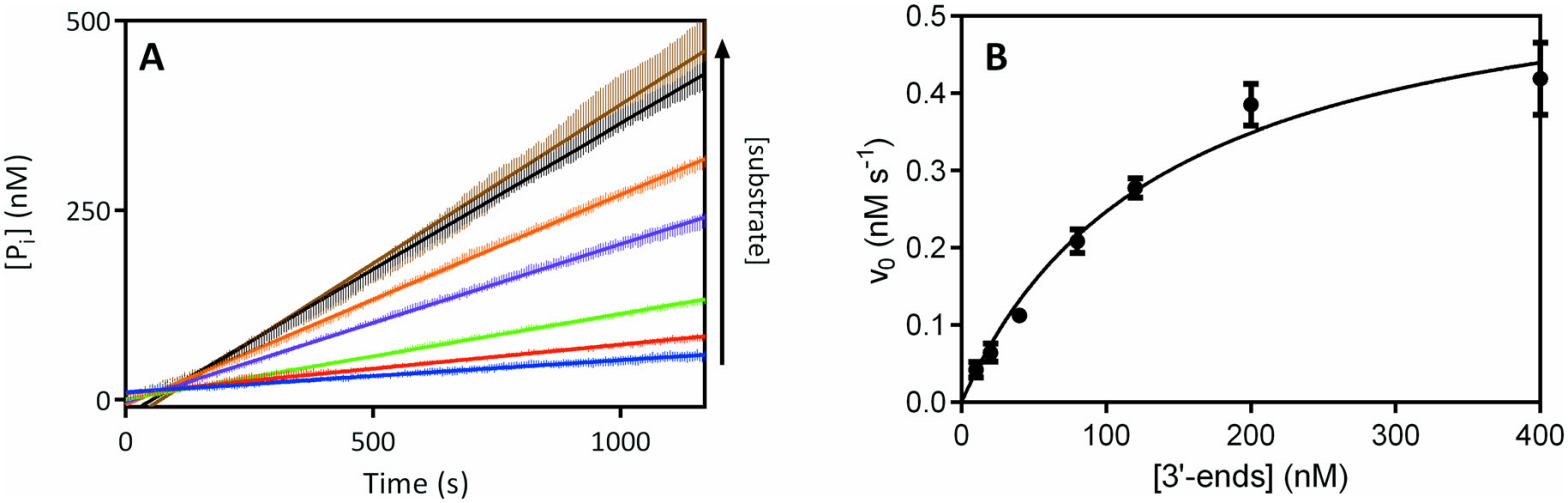
Exonuclease III steady state kinetics with increasing concentration of substrate by phosphate release assay. 1.5 nM ExoIII was incubated with 1μM MDCC-PBP, 0.0004u/μl FastAP and varying concentrations (5, 10, 20, 40, 60, 100 and 200 nM) of dsDNA substrate in 66mM Tris-HCl (pH 8.0) and 0.66mM MgCl_2_ at 37°C. (A) Fluorescence increase was measured over time from the ExoIII reaction coupled to FastAP dephosphorylation of products and subsequent P_i_ binding to MDCC-PBP. Background measured in parallel of a reaction without enzyme was subtracted from each data set. Fluorescence increase was converted to [P_i_] by interpolation from standard curve (S5 Fig). Data points are shown as bars of standard error of the mean of three independent experiments. Arrow (↑) refers to the increasing concentration of substrate in the different data sets. (B) Michaelis-Menten saturation curve by plotting initial velocity (v_0_) obtained from (A) against 3’-end concentration. Constants derived from plot were V_max_ = 0.5947 ± 0.0380 nM s^-1^ and K_M_ = 140.9 ± 20.3 nM.

ExoIII was used to evaluate the applicability of the assay for exonucleases [23]. ExoIII binds to blunt or recessed 3’ ends of dsDNA, regardless if the 3’ is phosphorylated or not, cleaving nucleotides from the 3’ end yielding deoxyribonucleoside-5’-monophosphates (dNMPs). Usually described as a processive exonuclease, ExoIII becomes more nonprocessive at higher temperatures (such as 37°C which is used here) [24]. FastAP, present at an excess over substrate, dephosphorylates the dNMPs for direct quantification by MDCC-PBP. Fig 1 depicts a kinetic model of the cleavage of dsDNA by ExoIII and subsequent P_i_-release by FastAP for MDCC-PBP P_i_ quantification [25]. P_i_ association to MDCC-PBP was determined as >200 nM s^-1^ in this setup (S2 Fig) and with a previously published association rate constant of 1.36 · 10^8^ M^-1^ s^-1^ was considered negligible to the overall rate of the phosphatase-coupled reaction [19]. k_on_ and k_off_ were not determined in this work. Since ExoIII is mostly a nonprocessive exonuclease at 37°C the enzyme will either move along the substrate without dissociating or dissociate and associate before cleavage of another nucleotide. As nucleotides are cleaved and released one at a time as dNMPs each event will have a different turnover numbers (k_cat (*n*)_) since ExoIII shows some sequence specificity [26]. Thus, an average of all k_cat (*n*)_ is measured and presented as ${\text{k}}_{\text{cat}}^{\text{app}}$. The experiment was repeated three times and as seen in Fig 2A the variation between each experiments is low and the reaction can easily be followed in real-time. The initial velocity data retrieved from Fig 2A was plotted against the concentration of dsDNA 3’-ends in Fig 2B and by using Michaelis-Menten kinetics we get a ${\text{k}}_{\text{cat}}^{\text{app}}$ value of 0.40 ± 0.025 s^-1^ and a K_M_ value of 140.9 ± 20.3 nM. ${\text{k}}_{\text{cat}}^{\text{app}}$ is determined by the amount of dNMPs produced in the reaction, which to our knowledge have never been measured for ExoIII before. K_M_ is determined using the concentration of 3’-ends as this serves as the binding site of the enzyme and the value determined (140.9 nM) is similar to previously published values (120 nM) [27]. The influence of FastAP dephosphorylation reaction rate on the overall reaction was negligible, determined in S3 Fig with a reaction rate more than 7 times the V_max_ of ExoIII.

### DNAzyme kinetics

To show the versatility of this assay a ‘10–23’ DNAzyme was employed; ExoIII being a protein-based exonuclease forming 5’-phosphorylated products compared to DNAzyme which endonucleolytically cleave their target once, forming 2’, 3’-cyclic phosphorylated products. DNAzymes have been used for gene knockdown, biosensors, molecular machines and many other applications. With the wide range of application for DNAzymes there is need for quick and robust kinetic assays. Several methods have previously been reported; they usually have drawbacks such as discontinuity, substrate has to be modified, can’t measure multiple turnovers and/or are not high-throughput. By using the assay presented here, multiple turnover reactions of a ‘10–23’ DNAzyme (eg. DzSJ) could be monitored in real-time.

**Fig 3:**
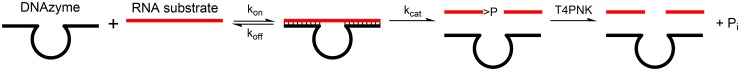
Kinetic model of DNAzyme reaction resulting in a product with a 2’,3’-cyclic phosphate (>P).

DNAzyme cleavage reaction yields a 2’, 3’-cyclic phosphate on one of the products, a form of organic phosphate that can’t be hydrolyzed by alkaline phosphatase. Instead an excess of T4PNK over substrate was used to dephosphorylate the product. Even though T4PNK is defined as a kinase it also has 3’ phosphatase activity with the ability to dephosphorylate 2’, 3’-cyclic phosphate ends. In Fig 3 the DNAzyme cleavage of an RNA substrate strand is shown, producing a cyclic phosphate, which is subsequently released as P_i_ by T4PNK for quantification by MDCC-PBP.

**Fig 4:**
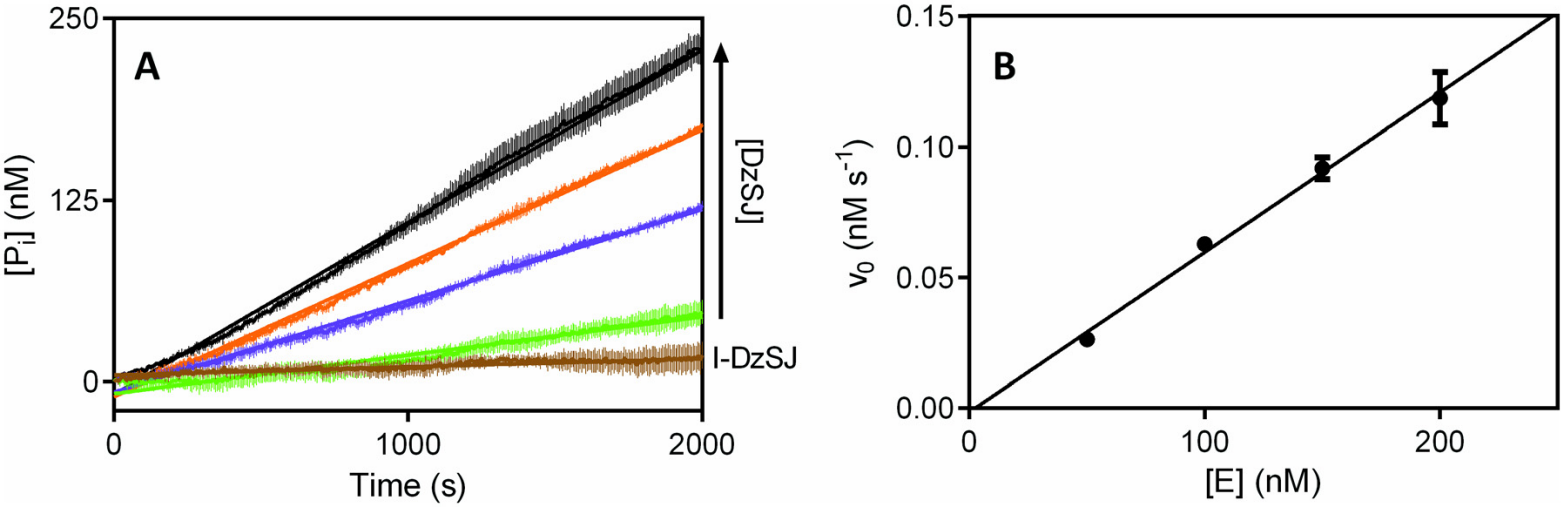
Phosphate release assay for ‘10–23’ DNAzyme steady state kinetics. Reactions were set up containing 2 μM RNA substrate, 1 μM MDCC-PBP, 0.3u/μl T4PNK and varying concentrations (50, 100, 150 and 200 nM) of DzSJ in 40 mM Tris-HCl (pH 7.5), 20 mM MgCl2 and 50 mM NaCl at room temperature. As a control 200 nM inactive DzSJ was used instead of DzSJ. Cleavage of RNA substrate exposes a 2’, 3’-cyclic phosphate (>P). The cyclic phosphate is released by T4PNK and quantified by fluorescence increase caused by P_i_ binding to MDCC-PBP. Background measured in parallel of a reaction without enzyme was subtracted from each data set. Fluorescence increase was converted to [P_i_] by interpolation from standard curve (S6 Fig). (A) Inorganic phosphate concentration plotted over time. Data points are shown as bars of standard error of the mean of three independent experiments. Arrow (↑) refers to the increasing concentration of DzSJ in the different data sets. (B) Initial velocities (v_0_) obtained from (A) plotted against DzSJ concentration showing a linear relation. R^2^ = 0.98.

The extremely low K_M_ of DzSJ (<1 nM) [20] is slightly lower than the limit of detection for this assay, hence the Michaelis-Menten equation could not be applied in this case. Instead different concentrations of DzSJ were used to show the linear increase of velocity with enzyme concentration, as can be seen in Fig 4. DzSJ is capable of multiple turnover catalysis which is shown in Fig 4A as the amount of P_i_ increased linearly over the monitored 2000 s and reached concentrations higher than the DzSJ concentration. Each initial velocity was determined by curve fitting and plotted against enzyme concentration in Fig 4B to show the linearity of velocity increase as the enzyme concentration is increased. With an R^2^-value of 0.98 the assay gives an excellent response to the enzyme increase and validates the versatility and robustness of the assay. A G-C mutation of the 6^th^ nucleotide in the catalytic loop of DzSJ served as a control for the experiment, showing no increase in fluorescence over the 2000 s time-course (Fig 4A). As T4PNK might influence the reaction rate of the overall reaction the dephosphorylation rate was determined in S4 Fig as being more than 4 times the highest rate measured for the DzSJ catalyzed cleavage. The perfect linearity of enzyme concentration dependence of the DzSJ cleavage reaction shown in Fig 4B also indicates T4PNK did not limit the overall reaction when high concentration of enzyme was used.

## Conclusions

The real-time microplate assay presented in this study proved to be highly versatile in the selection of nuclease, with low variability between experimental repeats. Protein, non-protein, exo- and endo- nucleases are assayed equally well with this assay. Comparison and exact determination of kinetics of nuclease catalysis can be easily performed with this assay, as well as mutation analysis and inhibitor screenings. As the need for labels is removed this assay will minimize the work for optimizing modifications of substrates and the cost of synthesis.

## Supporting Information

S1 Fig Determination of maximal background PNPase concentration. Different concentrations of PNPase 0.02 (blue), 0.01 (red), 0.002 (yellow), 0.001 (purple), 0.0002 (orange) and 0.00001 (black) u/μl was incubated with 1 μM MDCC-PBP and 200 μM 7-MEG in 40 mM Tris-HCl (pH 7.5) and 50 mM NaCl for 30 minutes at 37°C for temperature equilibration. After incubation measurements were done at an interval of 60 s over 10800 s with an addition of 175 nM P_i_ at 60 s. PNPase reacts P_i_ with 7-MEG forming ribose-1-phosphate which is not recognized by MDCC-PBP. Green line is the fitted straight line of 0.0002 u/μl PNPase with a slope of -0.002 nM s^-1^.
(TIF)

S2 Fig P_i_ association with MDCC-PBP. A solution of 1μM MDCC-PBP in 66mM Tris-HCl (pH 8.0) and 0.66mM MgCl_2_ at room temperature was measured every 0.25 s. Solutions of different concentration of KH_2_PO_4_ were added by Flexstation II fluidics module to the wells after 20 s, indicated by (↑), to measure association of P_i_ to the MDCC-PBP. Complete association at all concentrations can be seen within 2.5 s. At these conditions the velocity at the highest concentration of P_i_ was estimated to >200 nM s^-1^.
(TIF)

S3 Fig Dephosphorylation of ExoIII reaction products by FastAP. 1.5 nM ExoIII was incubated with 1μM MDCC-PBP, and 200 nM of dsDNA substrate in 66mM Tris-HCl (pH 8.0) and 0.66mM MgCl_2_ was incubated at 37°C for 20 min. The reactions were then measured every 2 s and 0.004 u/μl FastAP (red) or water (blue) was added after 27 s. Straight line curve fitting of the first 10% of the reaction is represented by the green line. Reaction velocity was calculated to 3.5 nM s^-1^.
(TIF)

S4 Fig Dephosphorylation of DzSJ reaction products by T4PNK. Reactions of 2 μM RNA substrate, 1 μM MDCC-PBP and 200 nM of DzSJ in 40 mM Tris-HCl (pH 7.5), 20 mM MgCl2 and 50 mM NaCl was incubated at room temperature for 30 min. The reactions were then measured every 1.3 s and 0.3 u/μl T4PNK (red) or water (blue) was added after 120 s. Straight line curve fitting of the first 10% of the reaction is represented by the green line. Reaction velocity was calculated to 0.56 nM s^-1^.
(TIF)

S5 Fig Standard curve for ExoIII reactions. 1 μM MDCC-PBP and varying concentrations of KH_2_PO_4_ in 66mM Tris-HCl (pH 8.0) and 0.66mM MgCl_2_ was incubated at 37°C for 20 min. The concentration of P_i_ is plotted against relative fluorescence units (RFU). Straight line curve fitting gives a slope equal to 899.2 ± 59.54 RFU nM^-1^. The R^2^-value of the curve straight line fitting was 0.98.
(TIF)

S6 Fig Standard curve for DzSJ reactions. 1 μM MDCC-PBP and varying concentrations of KH_2_PO_4_ in 40 mM Tris-HCl (pH 7.5), 20 mM MgCl2 and 50 mM NaCl was incubated at room temperature for 15 min. The concentration of P_i_ is plotted against relative fluorescence units (RFU). Straight line curve fitting gives a slope equal to 869.1 ± 40.32 RFU nM^-1^. The R^2^-value of the curve straight line fitting was 0.99.
(TIF)
