# Supplementary figures and images for: Quantitative Microplate Assay for Real-Time Nuclease Kinetics
Source: PLoS One. 2016 Apr 21;11(4):e0154099. doi: 10.1371/journal.pone.0154099 (PMC4839650; doi:10.1371/journal.pone.0154099)

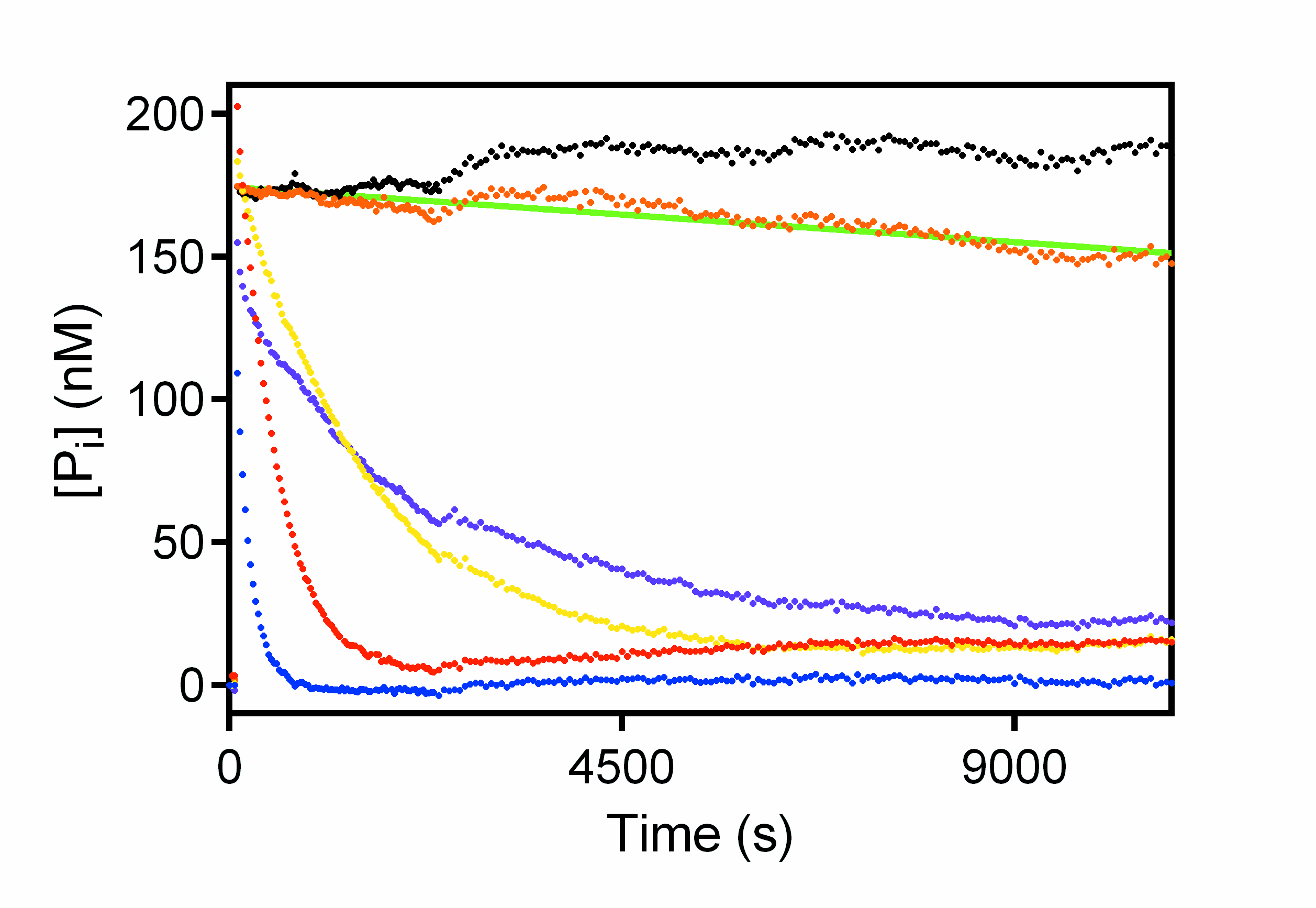

Supplement: S1 Fig — Different concentrations of PNPase 0.02 (blue), 0.01 (red), 0.002 (yellow), 0.001 (purple), 0.0002 (orange) and 0.00001 (black) u/μl was incubated with 1 μM MDCC-PBP and 200 μM 7-MEG in 40 mM Tris-HCl (pH 7.5) and 50 mM NaCl for 30 minutes at 37°C for temperature equilibration. After incubation measurements were done at an interval of 60 s over 10800 s with an addition of 175 nM Pi at 60 s. PNPase reacts Pi with 7-MEG forming ribose-1-phosphate which is not recognized by MDCC-PBP. Green line is the fitted straight line of 0.0002 u/μl PNPase with a slope of -0.002 nM s-1. (TIF) [file pone.0154099.s001.tif]

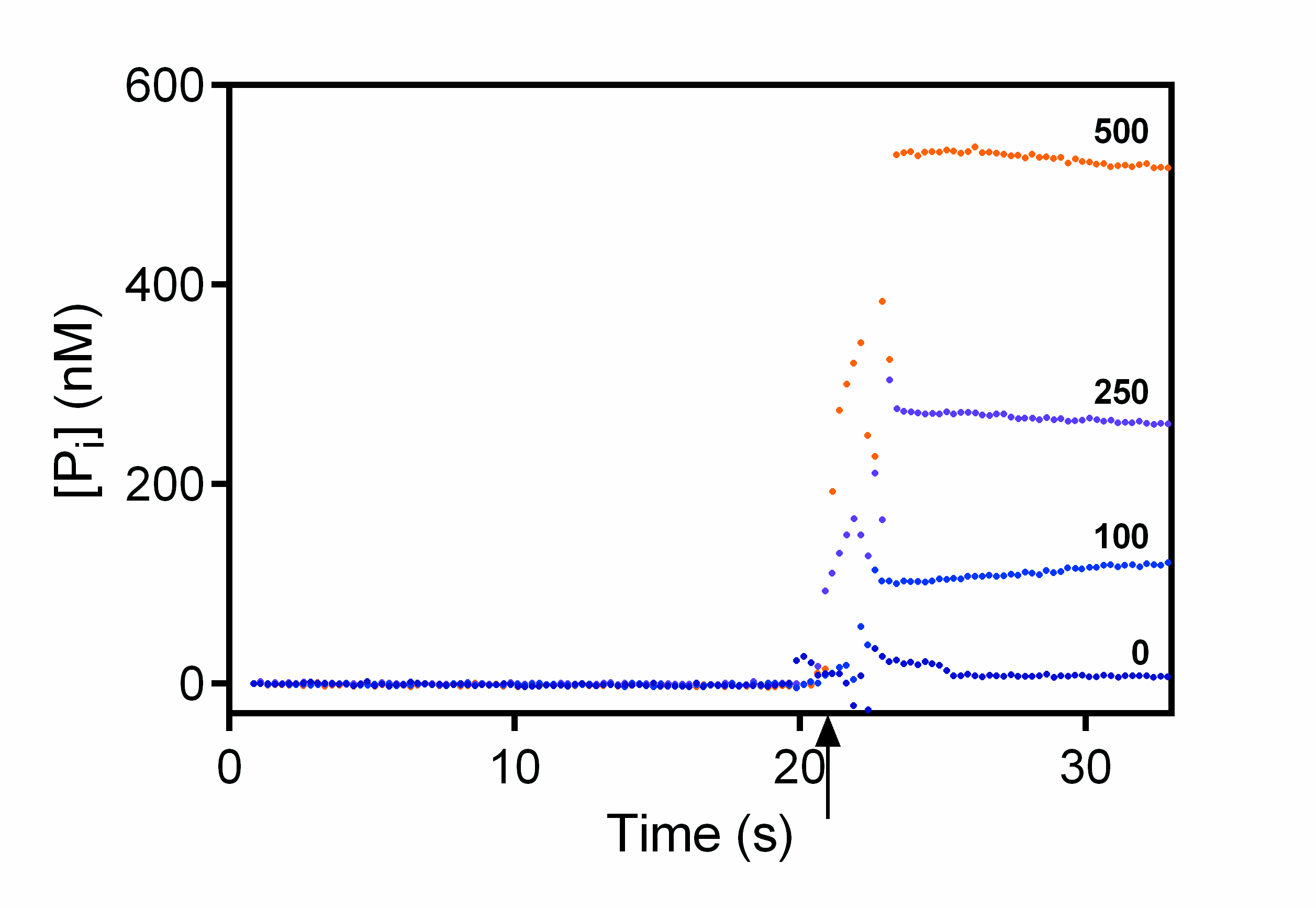

Supplement: S2 Fig — A solution of 1μM MDCC-PBP in 66mM Tris-HCl (pH 8.0) and 0.66mM MgCl2 at room temperature was measured every 0.25 s. Solutions of different concentration of KH2PO4 were added by Flexstation II fluidics module to the wells after 20 s, indicated by (↑), to measure association of Pi to the MDCC-PBP. Complete association at all concentrations can be seen within 2.5 s. At these conditions the velocity at the highest concentration of Pi was estimated to >200 nM s-1. (TIF) [file pone.0154099.s002.tif]

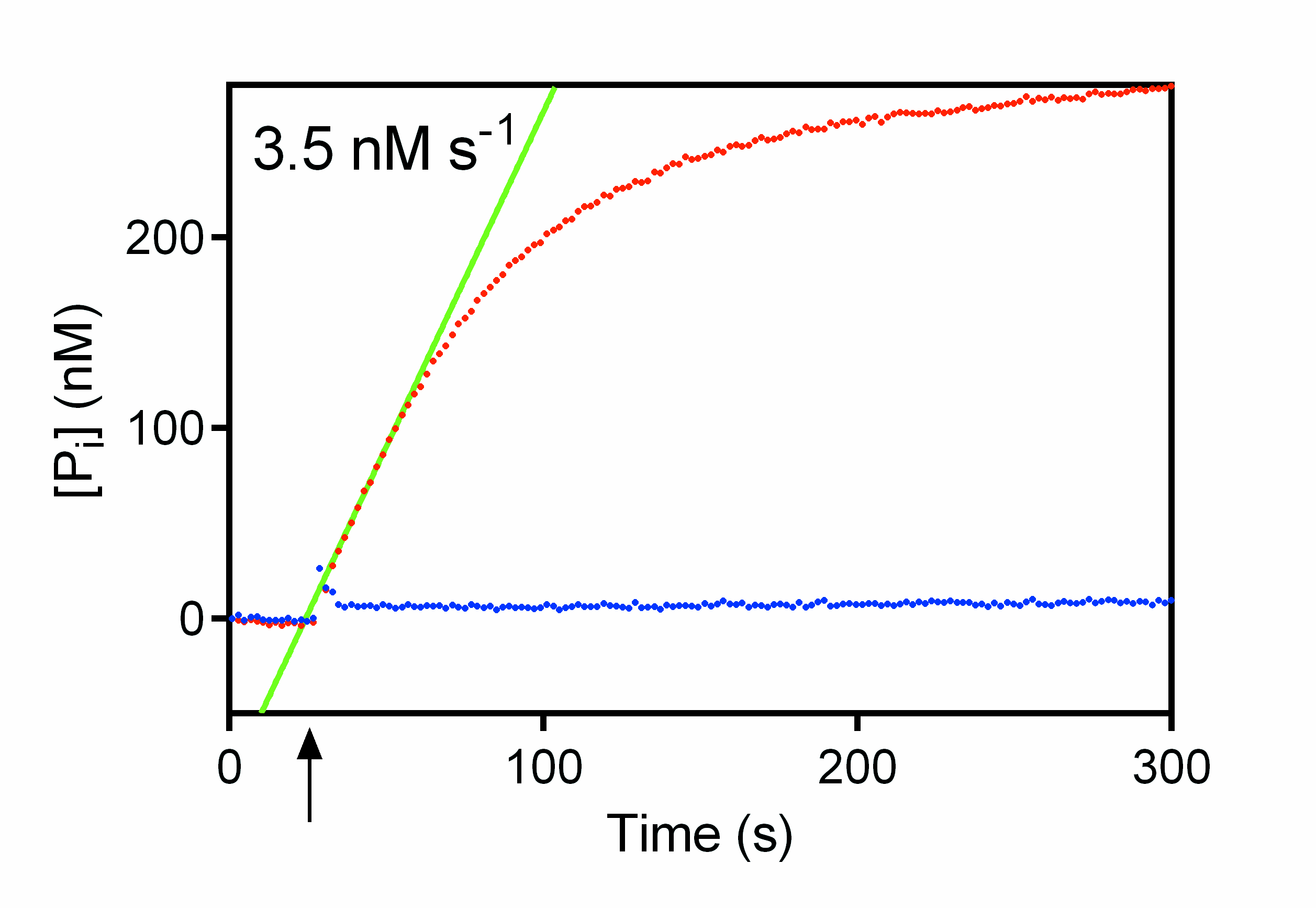

Supplement: S3 Fig — 1.5 nM ExoIII was incubated with 1μM MDCC-PBP, and 200 nM of dsDNA substrate in 66mM Tris-HCl (pH 8.0) and 0.66mM MgCl2 was incubated at 37°C for 20 min. The reactions were then measured every 2 s and 0.004 u/μl FastAP (red) or water (blue) was added after 27 s. Straight line curve fitting of the first 10% of the reaction is represented by the green line. Reaction velocity was calculated to 3.5 nM s-1. (TIF) [file pone.0154099.s003.tif]

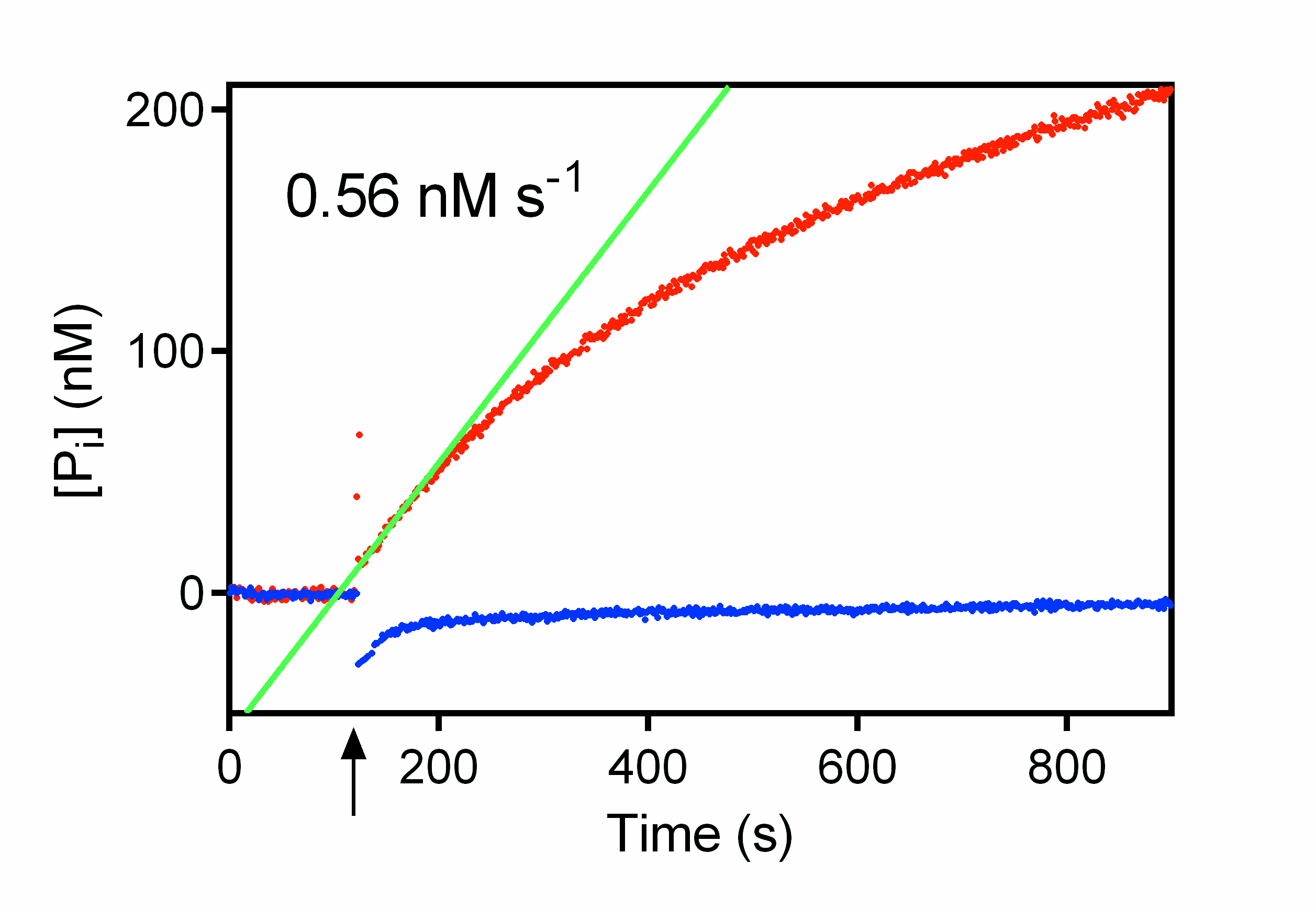

Supplement: S4 Fig — Reactions of 2 μM RNA substrate, 1 μM MDCC-PBP and 200 nM of DzSJ in 40 mM Tris-HCl (pH 7.5), 20 mM MgCl2 and 50 mM NaCl was incubated at room temperature for 30 min. The reactions were then measured every 1.3 s and 0.3 u/μl T4PNK (red) or water (blue) was added after 120 s. Straight line curve fitting of the first 10% of the reaction is represented by the green line. Reaction velocity was calculated to 0.56 nM s-1. (TIF) [file pone.0154099.s004.tif]

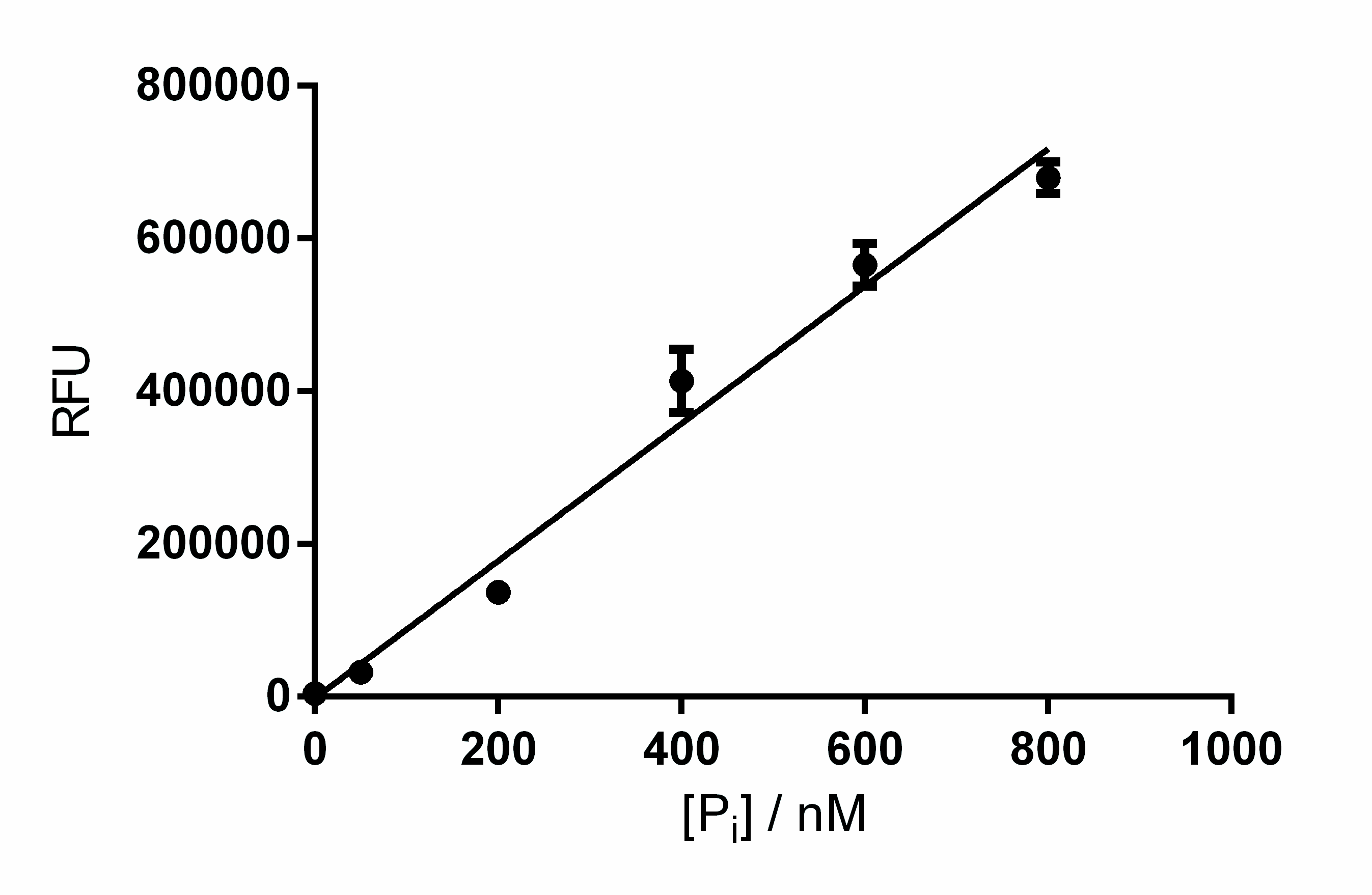

Supplement: S5 Fig — 1 μM MDCC-PBP and varying concentrations of KH2PO4 in 66mM Tris-HCl (pH 8.0) and 0.66mM MgCl2 was incubated at 37°C for 20 min. The concentration of Pi is plotted against relative fluorescence units (RFU). Straight line curve fitting gives a slope equal to 899.2 ± 59.54 RFU nM-1. The R2-value of the curve straight line fitting was 0.98. (TIF) [file pone.0154099.s005.tif]

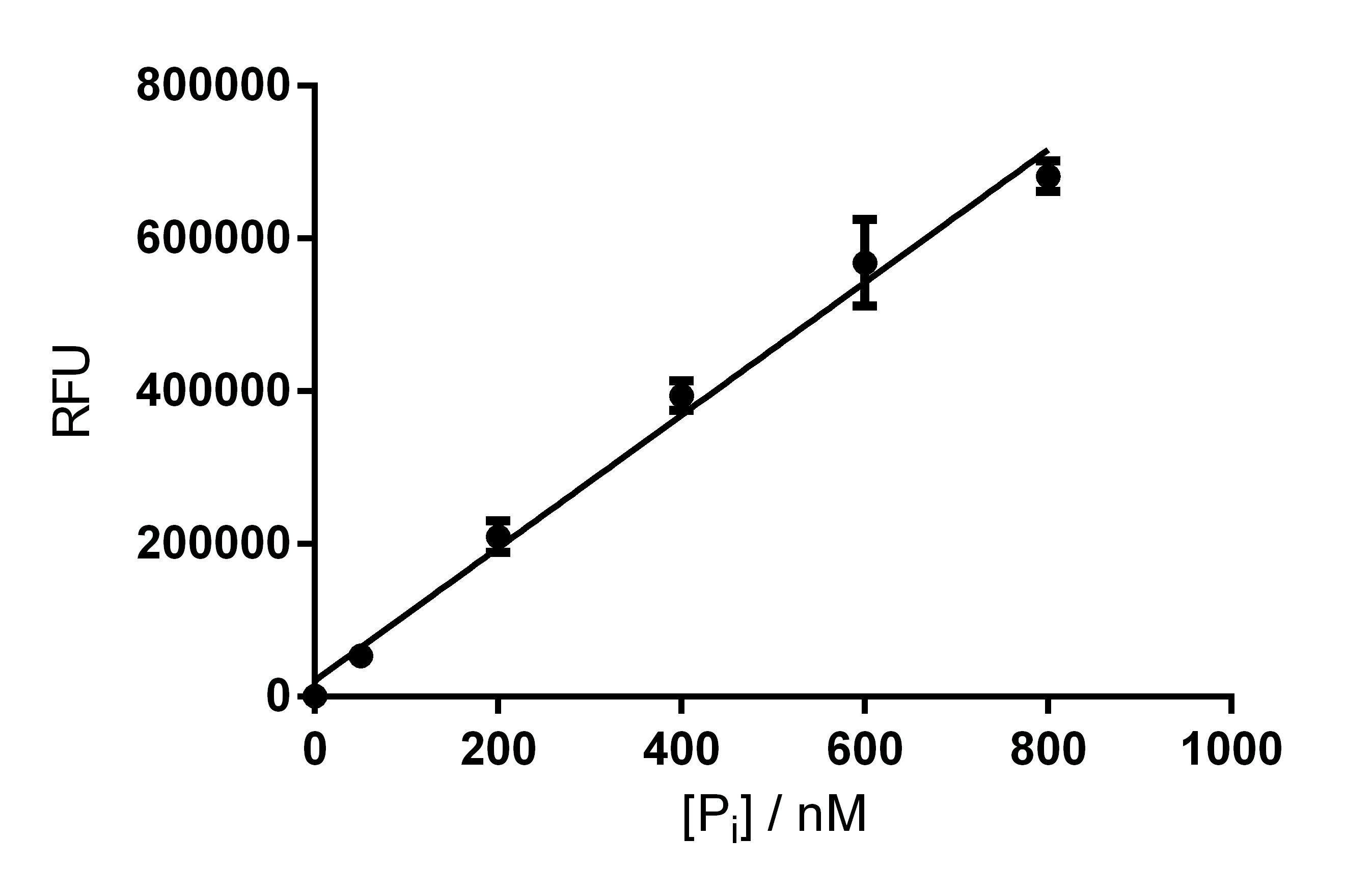

Supplement: S6 Fig — 1 μM MDCC-PBP and varying concentrations of KH2PO4 in 40 mM Tris-HCl (pH 7.5), 20 mM MgCl2 and 50 mM NaCl was incubated at room temperature for 15 min. The concentration of Pi is plotted against relative fluorescence units (RFU). Straight line curve fitting gives a slope equal to 869.1 ± 40.32 RFU nM-1. The R2-value of the curve straight line fitting was 0.99. (TIF) [file pone.0154099.s006.tif]
